# Characteristics and impact of physical activity interventions during substance use disorder treatment excluding tobacco: A systematic review

**DOI:** 10.1371/journal.pone.0283861

**Published:** 2023-04-26

**Authors:** Florence Piché, Catherine Daneau, Chantal Plourde, Stéphanie Girard, Ahmed Jérôme Romain

**Affiliations:** 1 Department of Human Kinetics, University of Quebec in Trois-Rivières, Trois-Rivières, Québec, Canada; 2 Faculty of Medicine, University of Montreal, Montreal, Québec, Canada; 3 Centre de recherche de l’Institut universitaire en santé mentale de Montréal, Québec, Canada; 4 Department of Psychoeducation, University of Quebec in Trois-Rivières, Trois-Rivières, Québec, Canada; Euro American University Center, BRAZIL

## Abstract

Substance use disorder is a worldwide issue that entails negative health and physical activity is a promising complementary therapy for alleviating the consequences. The objective of this reviews is to characterize physical activity interventions offered in the literature and explore their effects during treatment for people with substance use disorders with excluding studies focusing only on tobacco use. A systematic search of seven databases on articles including a physical activity intervention during a treatment for substance use disorder was done and an examination of the presence of bias was performed. A total of 43 articles including 3135 participants were identified. Most studies were randomized controlled trial (81%), followed by pre-post design (14%) and cohort studies (5%). The most common physical activity intervention identified was of moderate intensity, 3 times per week (≈ 1 hour) for 13 weeks. Cessation/reduction of substance use was the most studied outcome (21 studies, 49%), and 75% showed a decrease in substance use following physical activity intervention. Aerobic capacity was the second most studied effect (14 studies, 33%), with more than 71% of studies showing improvement. Twelve studies (28%) reported a decrease of depressive symptoms. Physical activity interventions in a treatment for substance use disorder seem to be a promising, but more methodologically rigorous scientific studies are needed.

## Introduction

Substance use disorder (SUD) is a worldwide issue with over 500 000 deaths per year [1], even if the proportion of people diagnosed per year with SUDs is minor (Worldwide: 2.3%; Canada: 4.2%) [2]. SUD is defined as a problematic substance use (including alcohol, cannabis, hallucinogens, phencyclidine, other hallucinogens, inhalants, opioids, sedatives; hypnotics; or anxiolytics, stimulants, tobacco, and other) despite the occurrence of cognitive, behavioral, and physical symptoms [3]. SUDs negatively affect various aspects of physical and mental health directly (intoxication, overdose, and misuse) and over the long term (cancer, heart disease, asthma, depression, anxiety disorder, etc.) [4,5]. Treatments for SUD (detoxification, residential settings, and outpatient facilities) [6] have poor adherence and important rate of relapse (50%) [7–9]. Thus, finding new ways to improve SUD treatment is a primary target for future research [10].

Physical activity (PA), defined as any body movement requiring energetic expenditure [11] has been recently considered as a therapeutic tool for people with serious mental illness (e.g., schizophrenia, major depressive disorder, bipolar disorder) considering its numerous benefits [12,13]. PA was found to improve depressive symptoms, cardiorespiratory fitness, quality of life and to not have adverse events compared to the control group [12,13]. For person with SUD, the only conclusive result is the reduction of craving symptoms in tobacco users by brief episodes of PA [14,15].

Several previous reviews on PA for people with SUD exist, but they have several limitations that prevent clear conclusions from being drawn: 1) most of the studies are on tobacco, and this is reflected in the results of reviews (e.g., 46–50% of the studies in reviews are exclusively on tobacco) [16,17] and that prevents from reaching conclusions regarding other substances. 2) When reviews focus on a single substance (e.g., alcohol) [18,19], this leads to the exclusion of studies that take into account poly users and leads to poor evidence and thus the impossibility of making clear conclusions through PA for SUD in general. It should be remembered that in clinical settings they are a large proportion of the people who had multiple SUD (e.g., for people with a cocaine use disorder, 60% have an alcohol use disorder, and 21% have a marijuana use disorder) [20]. 3) Reviews concentrate on outcomes exclusively related to SUD symptoms (e.g., craving, abstinence rate, withdrawal symptoms, quality of life, mood) [21,22]. This gives a limited view of the different possibilities of PA for people with SUD. Given the limited information available on the subject, it would be important to cover a wider range of outcomes. Knowing the impact on health in general could provide important additional information on the subject (e.g., breathing capacity, flexibility, sleep).

To overcome this gap in the literature, we conducted a systematic review of PA interventions for adults undergoing SUD treatment including all psychoactive substances except for studies focusing only on tobacco given the important body of knowledge on this substance. Our study was guided by the following two research questions: (1) What are the characteristics of PA interventions (frequency, duration, type, and intensity) during SUD treatment? (2) What are the physical, psychological, and social benefits of PA interventions during SUD treatment?

## Methods

The present systematic review was written following the Preferred reporting Items for systematic reviews and meta-analyses (PRISMA) statement [23]. The review was not registered and a protocol was not publish previously.

### Eligibility criteria

Studies were included in the present systematic review if they met the following PICOS inclusion criteria [23].

#### Population

Adults (≥18 years old) following a treatment for SUD related to psychoactive substances, including alcohol, cannabis, hallucinogens, phencyclidine, other hallucinogens, inhalants, opioids, sedatives; hypnotics; or anxiolytics, and stimulants. Studies only on tobacco use were excluded given there is already extensive published research on this topic [13,24].

#### Physical activity intervention

The PA intervention had to be done during in-person treatment (detoxification or residential). The intervention had to be a chronic (more than one PA session) PA intervention (sport, individual or group exercise). We excluded studies investigating the acute effects of PA and interventions that were not in person as this is associated with poorer retention for people with mental health disorders (e.g., television, video games, pamphlets, books) [25].

#### Comparison

A control group was not necessary for the objective of this present study.

#### Outcomes

All outcomes related to the impact of PA according to three categories: 1) physical as outcomes related to physical fitness and body composition (e.g., aerobic capacity, flexibility), 2) psychological as outcomes that takes into account mental life (e.g., depressive symptoms), and 3) life as outcomes that were related to the person’s behaviors and social environment.

#### Studies

Only experimental and observational studies having a quantitative design were included.

### Information sources

The following seven databases were searched from inception in July 2020 with an update in May 2022: CINAHL, Cochrane Library, PsycINFO, Medline, SCOPUS, SPORTDiscus and Google Scholar. We also screened the reference list of systematic reviews identified in the search, to identify more potential includable trials. Finally, we retained studies in English and French only.

### Search

To identify relevant studies and answer our research questions, we established keywords associated with three main concepts: PA, SUD and treatment. The search was conducted using keywords (see S1 Table) and Medical Subject Headings (Mesh) terms (see S2 Table) and they were:

a) for physical activity:

"Physical activity" OR "Sport" OR "Exercise" OR "Resistance Training" OR "Therapeutic Exercise" OR "Group Exercise" OR "Sport Specific Training".AND

b) for substance use disorder:

"Substance Abuse" OR "Substance Use Disorder" OR "Dependence" OR "Drug Abuse" OR "Addiction" OR "Morphine" OR "Heroin" OR "Opioid" OR "Opiate" OR "Cocaine" OR "Methadone" OR "Marijuana" OR "Cannabis" OR "Alcohol" OR "Drinker" OR "Methamphetamine" OR "Stimulant" OR "Substance Use Rehabilitation Programs" OR "Substance Dependence" OR "Narcotics" OR "Crack Cocaine" OR "Alcohol Rehabilitation Programs" OR "Alcoholism" OR "Alcohol Drinking".AND

c) for treatment:

"Inpatient Treatment" OR "Residential Treatment" OR "Long-Term Residential" OR "Addiction Center" OR "Detoxification".

#### Study selection

FP did the electronic search, and two authors (FP and CD) reviewed the result of the search independently and retained only those considered relevant according to the title and abstract. Full texts were checked for inclusion by the same two authors (FP and CD). The resulting articles were compared, and disagreement was resolved by discussion with a third researcher (SG).

#### Data collection process

Data were extracted independently by two authors (FP and CD) on a spreadsheet and comparison was made by FP between their observations to ensure their accuracy. If any disagreement between result was happening, it was resolved by discussion with a third researchers SG. Information was extracted for the study population (e.g., age, sex, diagnoses SUD criteria, substance use) and studies’ characteristics (e.g., design, type of control group, the number of participants in each group, the setting of the study). Also, outcome measures were collected (e.g., relevant statistical information) and PA interventions’ characteristics following the FITT method (frequency, intensity, time and type) [11].

### Risk of bias in individual studies

A quality analysis was conducted to obtain an overview of the different biases present in the selected studies. For this step, two researchers (FP and CD) rated the different studies independently and compared their results. Any disagreements on result were resolved by discussion with SG. We choose three ratings for the quality: *low risk*, *some concern*, and *high risk*. *Low risk* indicates a low risk of bias for all criteria, *some concern* indicates cause of concern regarding at least one criterion, and *high risk* indicates high risk regarding one criterion or substantial concerns regarding different criteria. To analyze the risk of bias, we used two pre-established grids selected according to recommendations in relation to the design of the study [26]. For the randomized controlled studies, we used the Cochrane risk-of-bias tool for randomized trials (RoB 2) [27]. The RoB 2 included five bias domains: 1. Randomization process; 2. Deviations from intended interventions; 3. Missing data; 4. Measurement of the outcome; 5. Selection of the reported result. For cohorts and pre-post design studies, we used the grids from the National Institutes of Health (NIH) study quality assessment tool [28] adapted for each design. The NIH tool for cohort and pre-post studies has up to 6 criteria for the quality of articles: 1. Research question; 2. Study population; 3. Sample size; 4. Exposure assessment or Intervention 5. Outcome measures; 6. Statistical analyses. Although it is not possible to compare these two grids (RoB and NIH tool) with each other, they can still provide us with valuable information on the biases present in the studies and therefore on how to improve future research.

## Results

### Selection of sources of evidence

**Fig 1:**
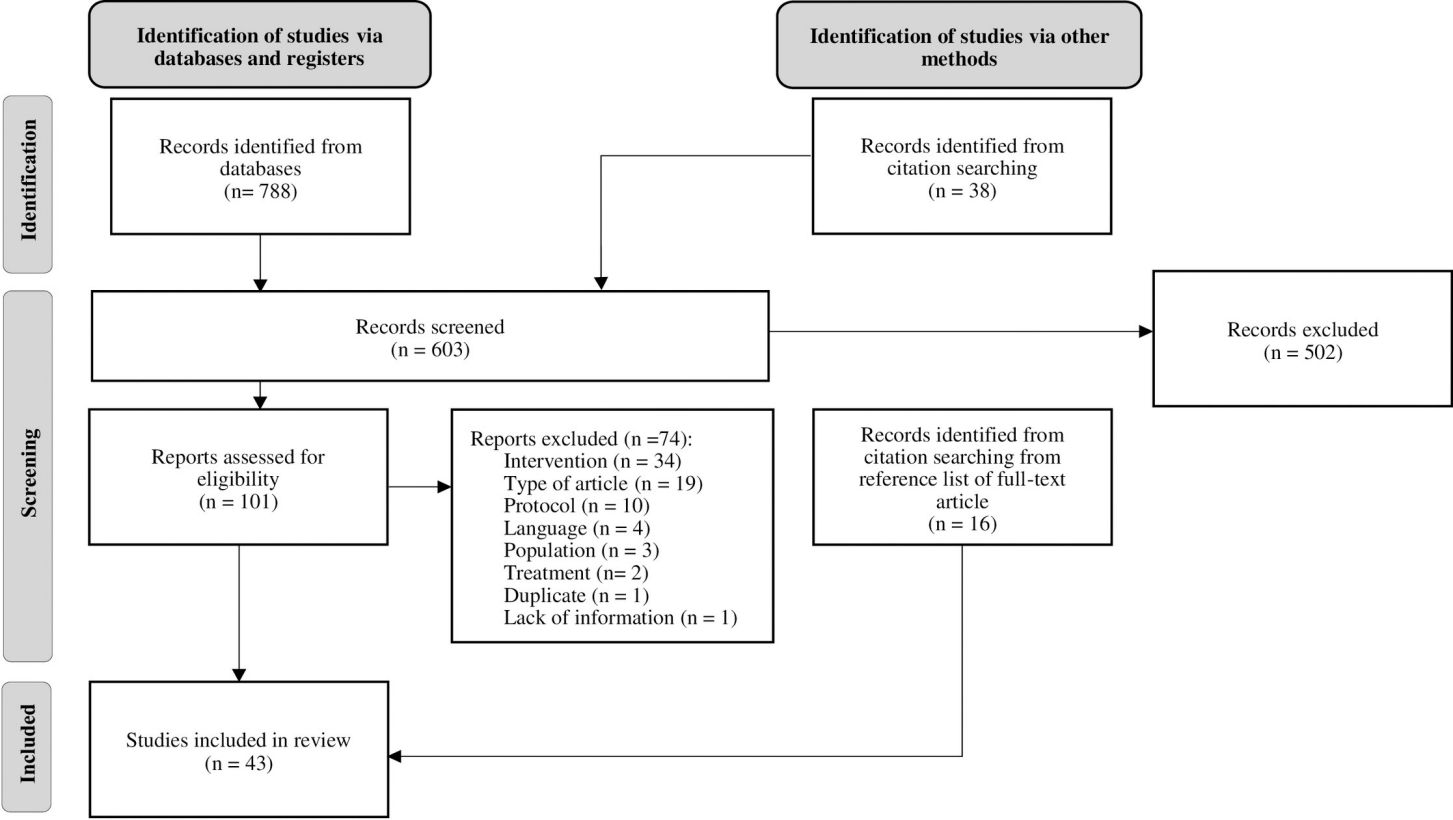
PRISMA flowchart.

After the initial search, 826 studies were identified (788 from databases and 38 from other sources); Following duplicate removal, 603 articles remained. After screening for titles and abstracts, 502 records were excluded because they did not meet the inclusion criteria (Fig 1). Next, 101 full texts were read and 74 were excluded (see Fig 1), which left with 27 studies. After screening the list of references of these 27 studies, 16 new articles were identified for a total of 43 [29–71].

### Characteristics of source of evidence and population

Most studies were published post-year 2000 (*K =* 37, 86%) and the remainder between 1972 and 1999 (*K =* 6, 14%). The majority were conducted in the United States (K = 17, 40%), followed by China (*K =* 11, 26%), Norway (*K =* 4, 9%) and Denmark (*K =* 2, 5%). The most popular design was the randomized controlled trial (*K =* 35, 81%), followed by pre-post design (*K =* 6, 14%) and cohort studies (*K =* 2, 5%). Most studies included both male and female patients (*K =* 25, 58%), but 23% only included men (*K =* 10) or 12% only women (*K =* 7), and 3 studies (7%) did not specify the sex. The total number of participants in all trials was 3135 (range 14–302). Regarding the diagnosis criteria, 17 studies (40%) were realized using the DSM (versions 4 and 5), and 6 (14%) according to the ICD-10: Classification of Mental and Behavioural disorders (F10-F19).

Regarding the intervention setting, 42% of the interventions took place in residences (*K =* 18), 28% in hospitals (*K =* 12) and 7% in clinics (*K =* 3); 23% did not specify the setting (*K =* 10). As regards to the type of substance treated, the most common was alcohol (*K =* 12, 28%), methamphetamine (*K =* 10, 23%), followed by all substances excluding nicotine (*K =* 10, 23%). The remaining articles (3 to 9%) focused only on amphetamine (*K =* 3), stimulants (*K =* 2), cocaine (*K =* 1), cannabis (*K =* 1), and heroin (*K =* 1). Otherwise, 7% (K = 3) only mentioned drugs as problematic substance. All the characteristics can be seen in S3 Table.

### Risk of bias

**Fig 2:**
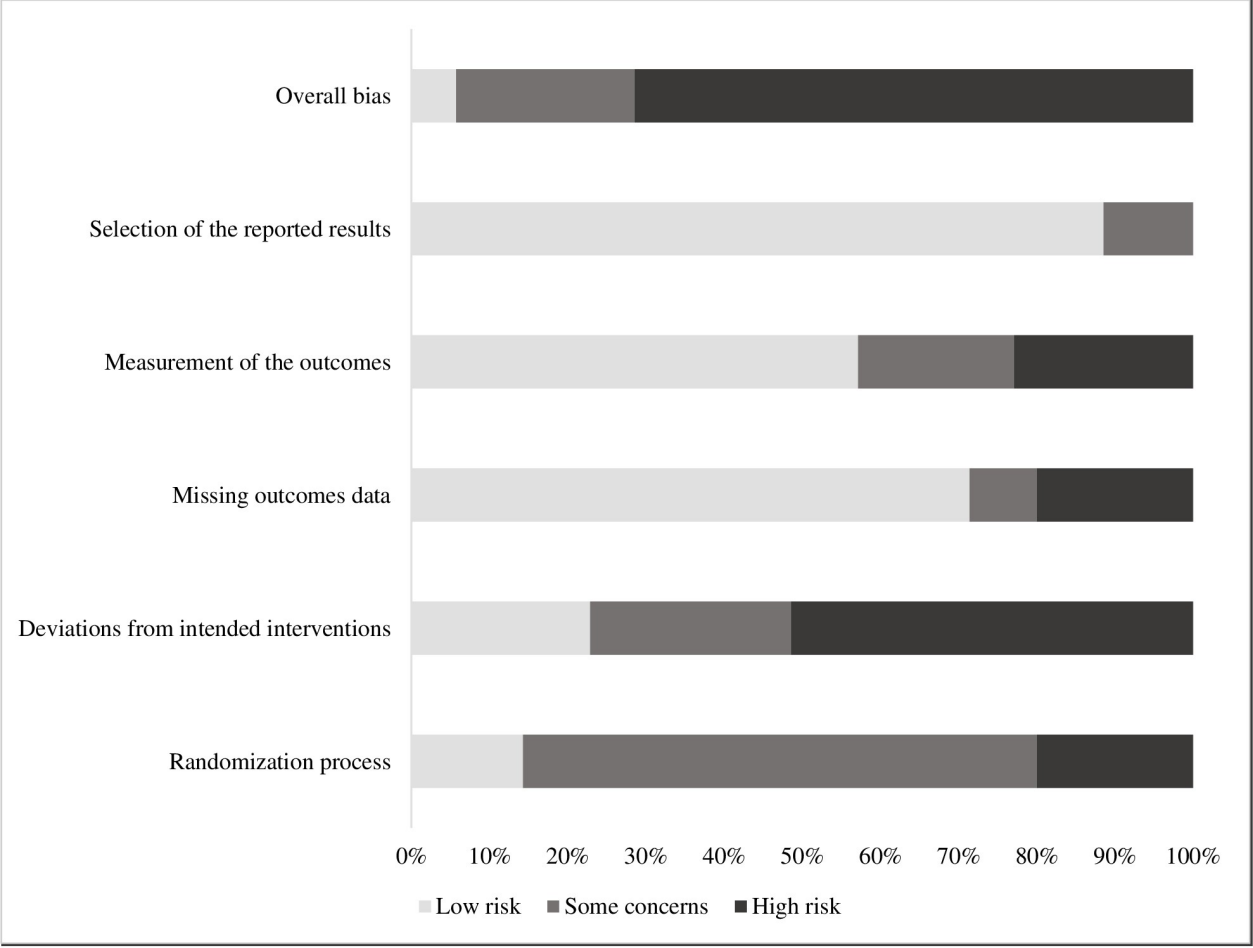
Risk of bias in randomized controlled trial studies using RoB 2.

Most studies (*K* = 28, 65%) coted high presence of bias regardless of study design (see S4, S5 and S6 Tables). Regarding risk of bias among included RCTs (Fig 2), the most important source was the deviation from intended interventions (50%). It is explained by the facts, studies poorly reported adherence to the intervention, or lost many participants during the intervention, or did not provide an appropriate analysis to balance the losses (intention-to-treat or modified intention-to-treat). Concerning the pre-post design and observational studies, three studies had *high* risk of bias because the population was not sufficiently described, six studies had a sample size too small, and for four studies the outcome was only measured once. Studies raised *some concerns* in terms of bias because the participation rate was below 50% (*K* = 16) and/or they failed to indicate if the assessor was blind to the intervention (*K* = 13).

### Physical activity intervention characteristics during SUD treatment

PA intervention characteristics are in (S7 Table). In terms of characteristics, the mean reported frequency of exercise was 3.4 ± 1.3 times a week followed (range 1–6). The mean intervention duration was 12.9 ± 10.7 weeks (range 1–52) and two studies did not indicate duration. The intensity of PA was unclear in many studies (*K =* 17, 40%). However, according to other information in the article (e.g., choice of activity, perceived rate, METS, percentage of VO_2_ max), 77% (*K =* 33) of interventions used moderate intensity, 21% light intensity (*K =* 9), 9% vigorous intensity (*K =* 4), 5% light and moderate intensity (*K =* 2), and 2% moderate and vigorous intensity (*K =* 1).

Jogging was the first choice of activity (*K =* 15, 35%) and it was combined with cycling (*K =* 4, 9%), resistance exercise (*K =* 3, 7%), elliptical training (*K =* 3, 7%), yoga (*K =* 2, 5%), jump rope (*K =* 1, 2%), ball games (*K =* 1, 2%) and walking (*K =* 1, 2%). The second most common activity was resistance exercise (*K =* 14, 33%) and it was also combined with aerobic (*K =* 5, 12%), cycling (*K =* 2, 5%) or an occasional sporting event (*K =* 2, 5%). Otherwise, the other reported activities were Tai chi (*K =* 4, 9%), yoga (*K =* 4, 9%), walking (*K =* 2, 5%), Taijiquan (*K =* 2, 5%), and softball (*K =* 1, 2%). The mean duration of PA was 51.7 ± 21.5 minutes (range 20–120). However, 28% of studies (*K =* 12) did not provide session duration of the PA training.

### PA outcomes during SUD treatment

#### Physical outcomes

**Fig 3:**
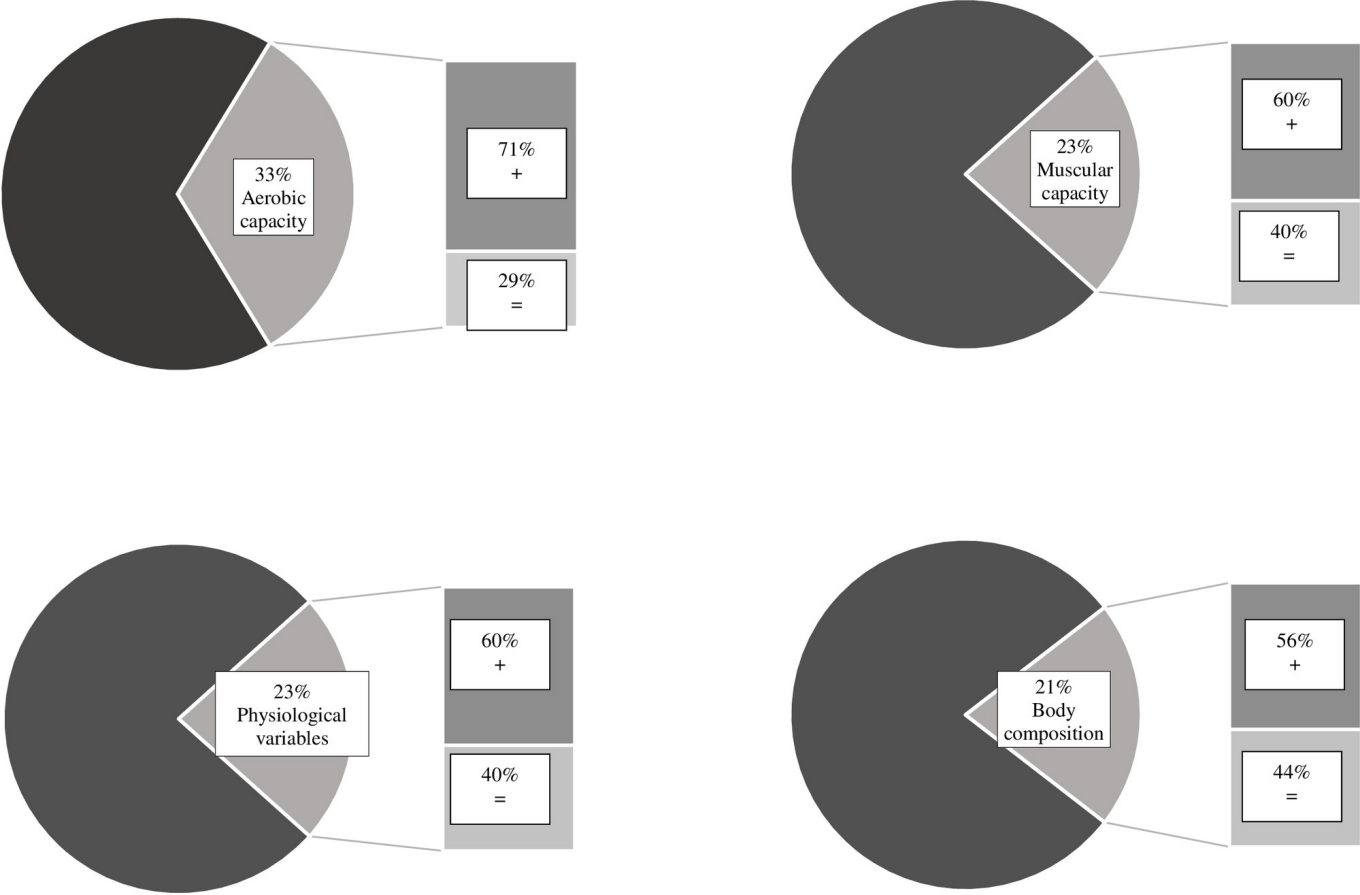
Physical outcomes. Each chart represents the percentage of studies that investigated the outcome out of the total number of studies and the bar on the side indicates the number of studies that saw improvement (+) and those that saw no change (=).

Over 63% of the studies included outcomes regarding physical fitness (*K =* 27; Fig 3). Aerobic capacity was the most studied outcome (*K =* 14, 33%), followed by muscular capacity (*K =* 10, 23%), which is a combination of muscle strength and endurance. The third most studied physical outcome was heart rate and blood pressure (*K =* 10, 23%). The fourth most measured outcome was body composition, including weight and body mass index; *K =* 9, 21%). The other outcomes were craving (*K =* 8, 19%), flexibility (*K =* 6, 14%), balance (K = 6; 14%) and a small number of studies took other outcomes into account: functional capacity (*K =* 1, 2%), skeletal health (*K =* 1, 2%), somatic health burden (*K =* 1, 2%), physical symptoms (*K =* 1, 2%), and withdrawal symptoms (*K =* 1, 2%). In the PA interventions, aerobic capacity improved significantly in 10/14 studies (71%), and muscular capacity in 6/10 studies (60%). For blood pressure, 3/10 studies (30%) found significant improvement and 6/10 for heart rate (60%). Also, improvement was found in 5/9 studies for body composition (56%), in 6/8 studies for craving (75%), in 3/6 studies for flexibility (50%) and in 4/6 (67%) studies for balance. The rest of outcomes was reported in one study: for physical symptoms, functional capacity and skeletal health, all studies reported significant improvement, and no change was observed for somatic health burden and withdrawal symptoms.

### Psychological outcomes

**Fig 4:**
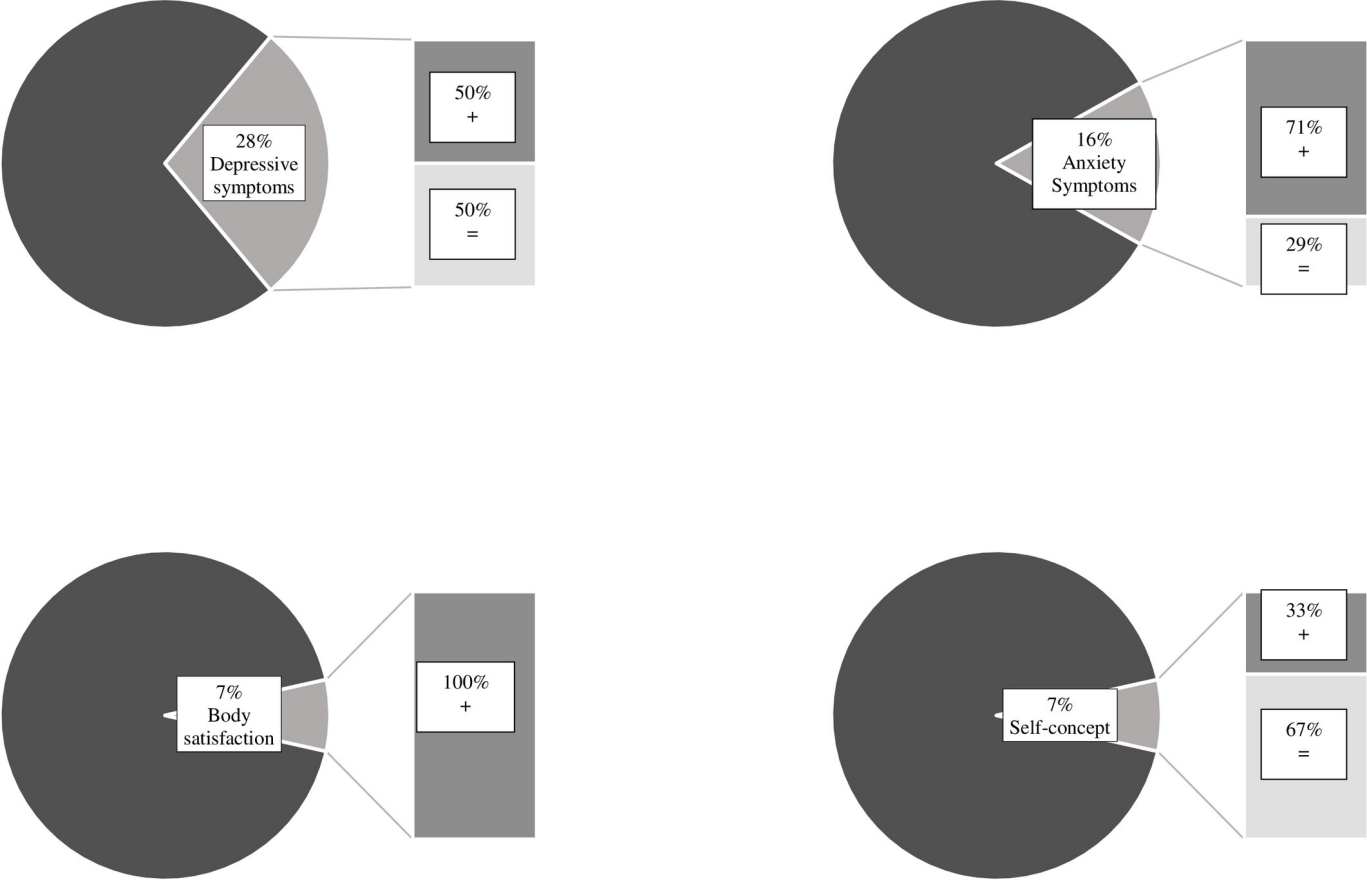
Psychological outcomes. Each chart represents the percentage of studies that investigated the outcome out of the total number of studies and the bar on the side indicates the number of studies that saw improvement (+) and those that saw no change (=).

Over 63% of the studies included psychological outcomes (*K =* 27; Fig 4). The studied psychological outcomes were depressive symptoms (*K =* 12, 28%), anxiety symptoms (*K =* 7, 16%), body satisfaction (*K* = 3, 7%), self-concept (*K =* 3, 7%), mood status (*K =* 2, 5%), working memory (*K =* 2, 5%), and executive function (*K =* 2, 5%). The remaining psychological outcomes were considered in a single study (2%); personality, brain activity, verbal memory, mindfulness, responsibility for health, inhibitory control, attention bias, cognitive function, mental distress and self-esteem. At the end of the intervention, significant improvements were found in 6/12 (50%) studies in depressive symptoms, and in 5/7 (71%) studies for anxiety symptoms. Body satisfaction and moods were significantly improved in all studies (3/3; 2/2). Otherwise, improvements were found in 1/3 of studies (33%) on the self-concept, and in 1/2 studies (50%) regarding executive function. Other remaining psychological outcomes (K = 1) showed significant improvement in attention bias, personality and responsibility for health, brain activity, and mindfulness. Otherwise, no effects were found regarding working memory, stress, inhibitory control, cognitive function, mental distress, verbal memory, and self-esteem were not statistically different.

### Life outcomes

**Fig 5:**
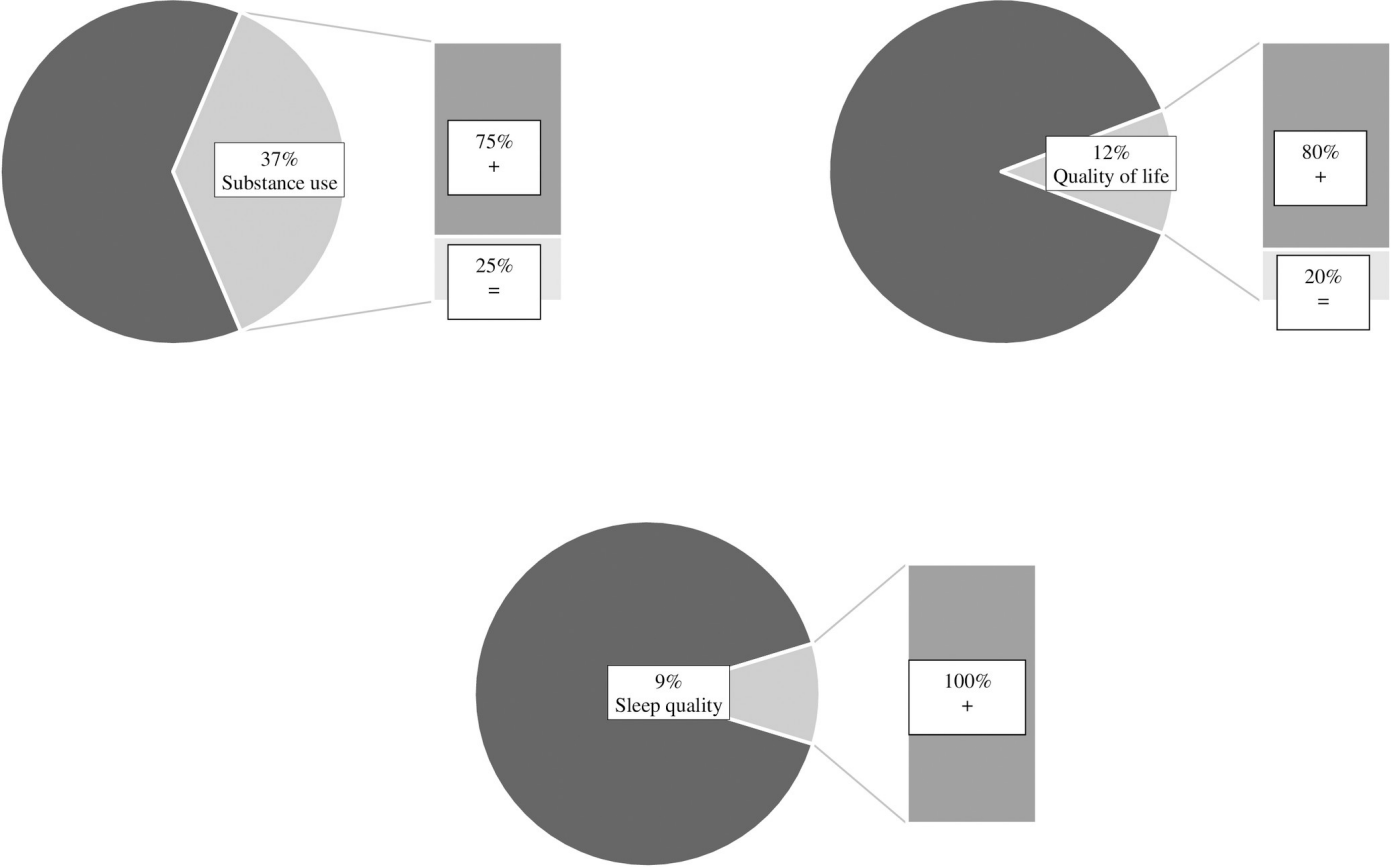
Life outcomes. Each chart represents the percentage of studies that investigated the outcome out of the total number of studies and the bar on the side indicates the number of studies that saw improvement (+) and those that saw no change (=).

Twenty-one studies considered life domains outcomes (49%; Fig 5). The most studied life outcome was substance use (*K =* 16, 37%), followed by health-related quality of life (*K =* 5, 12%) and sleep quality (*K =* 4, 9%). In terms of effects, 12 out of the 16 studies (75%) noted a significant decrease in substance use. Otherwise, improvements were found in all studies (4/4) on sleep quality, and in 4/5 studies (80%) for health-related quality of life. All the outcome, main result and bias are resume in S8 Table.

## Discussion

The objectives of the present systematic review were twofold. The first was to inform about the existing PA interventions during SUD treatments excluding study only on tobacco and to observe its effects on different outcomes, namely physical outcomes, psychological outcomes, and life outcomes. The most popular intervention was an exercise session duration of 60 minutes, of moderate intensity, three times a week, for 13 weeks and using jogging. The second objective was to better understand the effect of PA practice during SUD treatment. In this regard, the most discussed outcomes were aerobic capacity and muscular capacity. In terms of psychological outcomes, the two most studied with significant benefits were depressive and anxiety symptoms, respectively. For life outcomes, the most studied behavior was substance use followed by health-related quality of life and quality of sleep, all of which demonstrated significant improvement in most studies.

For the FIT (frequency, intensity, time), the characteristics found among included studies are quite classical (e.g., 2 to 3 times a week, the duration of 60 minutes for 13 weeks). Future studies should vary and access the different modalities of FIT to identify the optimal characteristics for the population undergoing treatment for SUD. The most reported intensities were moderate and/or vigorous, which may be explained by the fact that persons with SUD reported a preference towards moderate-intensity PA, followed closely by high-intensity exercise [72]. For the type, we found jogging and resistance exercise as the most common activities, a systematic review found that persons with SUD enjoy activities such as walking, resistance exercise and cycling [72]. Thus, as jogging was often coupled with walking this one activity and resistance exercise is in line with preferred activity observed in the literature for people with SUD [72]. We observed that cycling was used in 10 studies, and we believe it is because of the availability of equipment in the facility or hospital along with a low risk of injury. Also, yoga and tai chi also figured in nine studies. Given the literature shows that this activity attracts more women than men, it may be important to consider gender balance ratio in a facility for adherence to the activity [72]. Also, previous reviews underlined the potential role of yoga in the treatment of SUD [73,74] and suggest that this activity could reduce anxiety symptoms, pain, and substance use [74].

As regards to the second objective, three categories of outcomes were identified: physical, psychological and life domains. The mechanisms by which PA could decrease substance use are complex and operate in different levels [75]. At a physical level, increased awareness of one’s body, health and fitness may be a mechanism to help reduce alcohol and drug use [76]. Which is encouraging because most studies showed an increase in physical fitness, especially in aerobic capacity and muscle strength. Although extensive analyses like effect size have not been conducted, apart from blood pressure in physiological variables, it can be noted that most of the results show an improvement, which is in line with research in the general population [11]. Regarding the psychological outcomes, depressive symptoms and the presence of anxiety symptoms were the two most studied. The literature reveals that major depressive disorder or generalized anxiety disorder is very common in persons with SUD [77] and that PA is recommended to alleviate symptoms of depression and anxiety in the general population and in SUD for nicotine users [11,17,78]. Consequently, it is not surprising that most studies in our systematic review showed an improvement in the PA intervention. We also noticed that a change in anxiety symptoms was significantly associated with a change in symptoms of depression. In other words, when one of these outcomes improved, the other did so as well, possibly because there is often concomitance between these two [3]. Regarding life outcomes, substance use was the most studied outcomes, and it is because that abstinence or reduced consumption is often a major goal of treatment [79]. Most studies reported a reduction in substance use at the end of the treatment when PA was performed regardless of the substance. The way in which the studies investigated substance use is heterogeneous and this could possibly explain why some studies did not see differences in results. Still regarding life outcomes, the second outcomes were health-related quality of life and sleep quality. Most studies showed improvement for both, as is usually the case in the general population and this confirms what we already know on this topic [80,81].

Additionally, it is interesting that most studies (86%) did not mention adherence to PA, making it difficult to measure its impact on the participant and to compare one participant to another, because we don’t know what the actual dose of PA was received. This is also a very common bias in the studies: even those measuring adherence only provided an average proportion amount of participation levels for all the participants. No precise number or information was provided as to how it was included in the analysis. It would have been relevant to know the exact dose of PA received by each participant and to include it in the analyses. This would have made it possible to know, for example, what level of PA is required to perceive effects. It would also have allowed for a better comparison between the groups, knowing that sometime control groups (those without PA) may have included PA in their activities of daily living and that could have impact on result.

### Limitations

The strength of the present study is as follows: first, to the best of our knowledge, the present study is the first review examining PA as a part of treatment for people during a treatment for SUD for all substances excluding study on only tobacco and all types of PA; second, a quality assessment was conducted. Our study also had limitations. To begin, every culture is different and SUD treatments must be adjusted accordingly. A comparison of treatments is possible, but cultural differences should be considered. A major limitation is that many studies excluded persons with a mental comorbidity (e.g., bipolar disorder, schizophrenia, suicidal thoughts…), without providing a reason for this choice. This rationale is problematic given 60% of individuals with SUD suffer from an additional mental disorder s6]. The fact that many were excluded for this reason makes it very difficult to generalize results, as the study context does not represent real life. We suggest that future studies include all individuals with a mental comorbidity and examine them as a subgroup to highlight any differences. This will, of course, be necessary for future research. Another limitation is the fact that most of the studies have rated high in the risk of bias which leads us to have to relativize the conclusions found in this study and bring proposals to reduce it for future research.

## Conclusion

Our systematic review explores PA interventions during a treatment for persons with SUD. Results suggest that there is promising evidence indicating that PA can be beneficial for these patients. We conclude, too, that future researchers should better describe their interventions. We also maintain that it is important to consider including participants with mental comorbidities and to monitor PA adherence during the intervention and mention it in the results. This will help reduce methodological bias and allow for clear results that can be generalized.

## Supporting information

S1 Checklist PRISMA 2020 checklist. (DOCX)

S1 Table Keywords search strategy for each database. (PDF)

S2 Table MeSH terms used for search strategy. (PDF)

S3 Table Characteristics of included studies. (PDF)

S4 Table Characteristics of the physical activity interventions. (PDF)

S5 Table Outcomes measure, main results and bias. (PDF)

S6 Table Quality assessment tool for randomized trial (K = 35). (PDF)

S7 Table Quality assessment tool for observational cohort studies (K = 2). (PDF)

S8 Table Quality assessment tool for before-after (pre-post) studies with no control group (K *=* 6). (PDF)
