# Supplementary material for: Characteristics and impact of physical activity interventions during substance use disorder treatment excluding tobacco: A systematic review
Source: PLoS One. 2023 Apr 26;18(4):e0283861. doi: 10.1371/journal.pone.0283861 (PMC10132651; doi:10.1371/journal.pone.0283861)
Supplement: S1 Table — (PDF) [file pone.0283861.s002.pdf]

S1 Table. Keywords search strategy for each database.

Database	Keywords search
**CINAHL / APA PsycINFO / Sportdiscuss / Scopus / Medline / Cochrane**	("Physical activity" OR Sport* OR Exercise*) AND ("substance abuse" OR "Substance use disorder" OR Dependence OR "Drug abuse" OR addiction OR Morphine OR heroin OR opioid OR opiate* OR cocaine OR methadone OR marijuana OR Cannabis OR alcohol OR drinker OR methamphetamine OR stimulant) AND ("inpatient treatment" OR "residential treatment" OR "long-term residential" OR "addiction center" OR detoxification)
**Google Scholar**	"Physical activity" AND "Substance use disorder" AND "Addiction center" AND allintitle: Exercise "Drug abuse"
