# Supplementary material for: Characteristics and impact of physical activity interventions during substance use disorder treatment excluding tobacco: A systematic review
Source: PLoS One. 2023 Apr 26;18(4):e0283861. doi: 10.1371/journal.pone.0283861 (PMC10132651; doi:10.1371/journal.pone.0283861)
Supplement: S2 Table — (PDF) [file pone.0283861.s003.pdf]

S2 Table. MeSH terms used for search strategy.

Databases	MeSH terms for each keyword		
	**Physical activity**	**Substance use disorder**	**Treatment**
**CINAHL**	(MH "Physical Activity") OR (MH "Sports+") OR (MH "Exercise+") OR (MH "Resistance Training") OR (MH "Therapeutic Exercise") OR (MH "Group Exercise") OR (MH "Sport Specific Training")	(MH "Substance Abuse+") OR (MH "Substance Use Rehabilitation Programs+") OR (MH "Substance Dependence+") OR (MH "Substance Use Disorders+") OR (MH "Morphine+") OR (MH "Heroin") OR (MH "Narcotics+") OR (MH "Cocaine+") OR (MH "Crack Cocaine") OR (MH "Methadone") OR (MH "Cannabis") OR (MH "Alcohol Rehabilitation Programs+") OR (MH "Alcoholism") OR (MH "Alcohol Drinking+") OR (MH "Methamphetamine+")	n/a
**APA PsycINFO**	DE "Physical Activity" OR DE "Sports" OR DE "Exercise" OR DE "Aerobic Exercise"	DE "Drug Abuse" OR DE "Substance Abuse and Addiction Measures" OR DE "Substance Use Treatment" OR DE "Alcohol Treatment" OR DE "Substance Use Disorder" OR DE "Substance Related and Addictive Disorders" OR DE "Cannabis Use Disorder" OR DE "Opioid Use Disorder" OR DE "Alcohol Use Disorder" OR DE "Morphine Dependence" OR DE "Alcoholism" OR DE "Drug Addiction" OR DE "Drug Dependency" OR DE "Addiction" OR DE "Heroin Addiction" OR DE "Morphine" OR<br><br>DE "Heroin" OR "Opioid+" OR DE "Opiates" OR DE "Cocaine" OR DE "Crack Cocaine" OR	DE "Residential Care Institutions" OR DE "Rehabilitation Centers" OR DE "Detoxification"

		DE "Methadone" OR DE "Marijuana" OR DE "Cannabis Use Disorder" OR DE "Cannabis" OR<br><br>DE "Alcohol Abuse" OR DE "Methamphetamine"	
**Sportdiscuss**	DE "PHYSICAL activity" OR DE "EXERCISE" OR DE "RESISTANCE training" OR DE "INTERVAL training" OR DE "HIGH-intensity interval training" OR DE "SPORT for all" OR Exercise<br><br>+	DE "SUBSTANCE abuse" OR DE "DRUG abuse" OR DE "ADDICTIONS" OR DE "DRUG addiction" OR DE "MORPHINE" OR DE "HEROIN" OR DE "HEROIN abuse" OR DE "NARCOTICS" DE "OPIOID abuse" OR DE "OPIOIDS" OR DE "COCAINE" OR DE "COCAINE abuse" OR DE "CRACK cocaine" OR DE "MARIJUANA" OR DE "MARIJUANA abuse" OR DE "CANNABIS" OR DE "ALCOHOL" OR DE "ALCOHOL drinking" OR DE "ALCOHOLISM" OR DE "ALCOHOLICS" OR DE "METHAMPHETAMINE" OR DE "METHAMPHETAMINE abuse" OR DE "STIMULANTS"	DE "SUBSTANCE abuse treatment" OR DE "DETOXIFICATION (Substance abuse treatment)" OR DE "ALCOHOLISM treatment" OR DE "TREATMENT programs" OR DE "TREATMENT of addictions" OR Detoxification +
**Scopus**	N/A	N/A	N/A
**Medline**	(MH "Exercise+") OR (MH "Sports+") OR (MH "Water Sports")	(MH "Substance-Related Disorders+") OR (MH “Substance use disorder+”) OR (MH "Dependance+") OR (MH "Addiction+") OR (MH "Morphine") OR (MH "Morphine Dependence") OR (MH "Heroin") OR (MH "Heroin Dependence") OR<br><br>(MH "Opioid-Related Disorders") OR (MH "Opiate+") OR (MH "Cocaine") OR (MH	(MH "Inpatients") OR (MH "Residential Treatment") OR (MH "Long-Term Care") OR<br><br>(MH "Community Health Centers") OR (MH "Rehabilitation Centers") OR (MH "Metabolic Detoxication, Phase II") OR (MH "Metabolic Detoxication, Phase I")

		"Crack Cocaine") OR (MH "Cocaine-Related Disorders") OR (MH "Methadone") OR (MH "Marijuana Use") OR (MH "Marijuana Abuse") OR (MH "Marijuana Smoking") OR (MH "Cannabis") OR (MH "Alcohol Drinking") OR (MH "Alcohol Abstinence") OR (MH "Drinker+") OR (MH "Methamphetamine")	
**Cochrane**	MeSH descriptor: [Exercise] explode all trees OR MeSH descriptor: [Sports] explode all trees	MeSH descriptor: [Substance-Related Disorders] explode all trees OR MeSH descriptor: [Morphine] explode all trees OR MeSH descriptor: [Opioid-Related Disorders] explode all trees OR MeSH descriptor: [Cocaine] explode all trees OR MeSH descriptor: [Methadone] explode all trees OR MeSH descriptor: [Cannabis] explode all trees OR MeSH descriptor: [Alcohols] explode all trees OR MeSH descriptor: [Methamphetamine] explode all trees	MeSH descriptor: [Residential Treatment] explode all trees OR MeSH descriptor: [Long-Term Care] explode all trees OR MeSH descriptor: [Rehabilitation Centers] explode all trees
**Google Scholar**	N/A	N/A	N/A
