# Supplementary material for: Characteristics and impact of physical activity interventions during substance use disorder treatment excluding tobacco: A systematic review
Source: PLoS One. 2023 Apr 26;18(4):e0283861. doi: 10.1371/journal.pone.0283861 (PMC10132651; doi:10.1371/journal.pone.0283861)
Supplement: S3 Table — (PDF) [file pone.0283861.s004.pdf]

S3 Table. Characteristics of included studies.

Author, year, country	Study Design	Study population			SUD criteria	Setting	Substance	Study aims
		Total *N*	*N* Female	Comparison(s)/ Control(s)				
**Abatti Martins et al. (2017)**<br>**Brazil**	*RCT	*N* = 20	N/A	*N* = 10	a) Crack as the primary reason for admission, b) use of this substance for six months minimum, c) of greater or equal hospital stay than 90 days.	Hospital	Crack cocaine	To investigate the effects of resistance exercise on the anthropometric parameters and components of physical fitness in ex-crack cocaine users.
**Brown et al. (2009)**<br>**USA**	Pre-post	*N* = 19	*N* = 11	N/A	a) met current DSM-IV criteria for drug dependence.	Outpatient	Alcohol	The current study is intended to address this need by describing the development of a moderate intensity aerobic exercise program for alcoholics.
**Brown et al. (2010)**<br>**USA**	Pre-post	*N* = 16	*N* = 5	N/A	a) met current DSM-IV criteria for drug dependence.	Inpatient and outpatient	All	To examine the feasibility and exercise adherence among drug-dependent patients.<br>To examine drug use and cardiorespiratory fitness outcomes at the end of the 12-

								week intervention and at the 3-month follow-up.
**Brown et al. (2014)**<br>**USA**	RCT	*N* = 49	*N* = 22	*N* = 23	a) meeting DSM-IV-TR criteria for alcohol dependence	Hospital	Alcohol	We aimed to test the efficacy of an aerobic exercise intervention as an adjunct treatment in reducing alcohol use among physically sedentary alcohol dependent patients in early recovery.
**Burling et al. (1992)**<br>**USA**	Cohort	*N* = 218	*N* = 1	*N* = 102 (TAU**)<br>N = 82 (1 year before)	a) Be in treatment for at least 30 days.	Residential	Alcohol and/or drug	To collect data systematically to see if these observations (team members seemed to have a more positive treatment outcome than non-members) could be empirically validated.
**Capodaglio et al. (2003)**<br>**Italy**	RCT	*N* = 74	*N* = 18	*N* = 23 (TAU)<br>*N* = 18 (healthy)	a) Be alcoholic dependence, as<br>defined by DSM IV,1 with habitual daily alcoholic<br>intake > 100 g.	-	Alcohol	To assess work capacity in patients with alcoholic dependence on working age and to monitor changes after an aerobic programme.

**Carmody et al. (2018)**<br><br>**USA**	RCT	*N* = 218	**-**	*N* = 143	a) Admitted to residential substance abuse treatment, b) had used stimulants within 30 days prior to enrollment, c) met DSM-IV criteria for stimulant abuse or dependence within the last 12 months.	Residential and outpatient	Stimulant	To determine the potential effectiveness of adequately dosed exercise for substance use disorder.
**Dolezal et al. (2013)**<br><br>**USA**	RCT	*N* = 29	*N* = 4	*N* = 14	a) Be in-residence at the treatment center, b) meet DSM-IV-TR criteria for MA dependence.	Residential	Methamphetamine	To assess the feasibility of a combined 8-week aerobic and resistance exercise training protocol in a sample of individuals undergoing residential treatment for methamphetamine dependence.
**Ermalinski et al. (1997)**<br><br>**USA**	RCT	*N* = 90	N/A	*N* = 42	-	Inpatient	Alcohol	To evaluate, in a true experimental paradigm, the impact of a body-oriented component in a group treatment program with hospitalized alcoholics.
**Fitzgerald et al. (2020)**	Cohort	*N* = 34	*N* = 17	N/A	-	Community	All	To gauge the impact of therapeutic yoga

**UK**								classes upon the mood state of recovering participants and explore the perceived benefits of therapeutic yoga class participation.
**Flemmen et al.** **(2014)** **Norway**	RCT	*N* = 16	*N* = 3	*N* = 7	a) Diagnosis of substance use disorder, ICD-10: F10-F19, b) be in a residential long-term treatment of a substance abuse treatment clinic.	Residential	Illegal drugs	To examine if high-intensity interval training was feasible for substance use disorders patients in treatment and to document their aerobic power and compare the training group, if they were able to adhere, with patients receiving conventional treatment in the same clinic.
**Frankel and Murphy (1974)** **USA**	Pre-post	*N* = 214	N/A	N/A	-	Hospital	Alcohol	To validate a new index of physical fitness to discover the extend and nature of the relationship between physical fitness and personality.
**Gaihre and Rajesh (2017)**	RCT	*N* = 87	-	*N* = 43	a) Meet the DSM-V criteria for	In-house	All	To evaluate the efficacy of a yoga-based intervention as

**India**					substance use disorder.			an add-on in enhancing cognitive functions, compared with physical exercise to newly admitted substance abusers seeking an inpatient treatment program.
**Gary and Guthrie (1972)**<br>**USA**	RCT	*N* = 36	-	-	-	Hospital	Alcohol	To improve physical condition could be expected to contribute to re-establishing counter-dependent defenses and reducing the pressure for continued alcoholic behavior.
**Giesen et al. (2016)**<br>**Germany**	RCT	*N* = 51	*N* = 14	*N* = 19<br>(TAU)<br>*N* = 18 (healthy)	a) Diagnosis of alcohol dependence (F10.2) according to the diagnosis criteria of the ICD-10, b) currently abstinent and housed in the residential facility.	Residential	Alcohol	To examine the feasibility of a severe alcohol use disorder specialized exercise program and to investigate if severe alcohol use disorders patients profited from this program related to their physical activity level and their health-related quality of life.
**Haglund et al. (2014)**	RCT	*N* = 135	*N* = 40	*N* = 66	a) MA dependence (by DSM-IV).	Residential	Methamphetamine	To investigate individual factors that

**USA**								predicted the most significant depression response to exercise therapy among our sample.
**Hallgren et al. (2014)**<br><br>**Sweden**	RCT	*N* = 14	-	*N* = 6	a) The DSM-IV criteria for alcohol dependence.	Outpatient	Alcohol	To briefly review the literature on yoga and alcohol dependence treatment, to assess the feasibility of incorporating yoga as part of treatment for alcohol dependence and to assess quantitative and qualitative approaches to study the outcome of this form of treatment.
**Li et al. (2013)**<br><br>**China**	RCT	*N* = 33	All	*N* = 16	a) Diagnosis of opioid dependence.	-	Opioid	To measure if Tai Chi had a pronounced effect on protracted abstinence syndrome.
**Liu et al. (2021)**<br><br>**China**	RCT	*N* = 98	All	*N* = 49	a) Having a positive methamphetamine urine test result, b) having received at least 3 months of therapy	Hospital	Methamphetamine	To explore the effects of an aerobic exercise program on cognitive function at two points in time: (a) immediately after completion of the exercise program and (b) at 3 months postintervention.

**Lu et al. (2020)**<br><br>**China**	RCT	*N* = 63	N/A	*N* = 21	a) Met the DSM-V criteria	Center	Methamphetamine	To evaluate and compare the effects of aerobic exercise and anaerobic exercise on craving and dynamic brain states for methamphetamine patients.
**McCartney et al. (2020)**<br><br>**Australia**	RCT	*N* = 46	*N* = 13	*N* = 22	a) ICD-10	Hospital	Cannabis	To examine the effect of six, once-daily, bouts of aerobic cycling exercise on subjective and objective sleep quality metrics during inpatient cannabis withdrawal in dependent cannabis users.
**Muller and Clausen (2015)**<br><br>**Norway**	Pre-post	*N* = 35	*N* = 26	N/A	a) Enrolled in long-term residential SUD treatment.	Residential	All	To explore the feasibility and quality of life effects of the group exercise among residential substance use disorder patients, through the implementation of a 10-week, low-intensity group exercise program that was voluntary and led by motivating coach,

								outside of the treatment system.
**Ness et al. (2001)**<br>**USA**	Pre-post	*N* = 13	*N* = 4	N/A	-	Inpatient	Alcohol	To determine the effects of a physical therapist designed strengthening and conditioning program on aerobic performance, strength, balance, and functional ability in individuals with chronic alcoholism who were receiving treatment in a long-term care facility.
**Nygard et al. (2018)**<br>**Norway**	RCT	*N* = 23	*N* = 6	*N* = 12	a) Diagnosis of SUD according to the WHO diagnostic system (ICD-10: F10–F19), b) having amphetamines as their main drug.	Residential	Amphetamine as main drug	To investigate the effects of hack-squat maximal strength training on skeletal muscle force generating capacity and skeletal health.
**Palmer et al. (1988)**<br>**USA**	RCT	*N* = 53	*N* = 16	*N* = 26	-	Inpatient	Alcohol	Address the above design weaknesses and to combine an examination of psychological variables with a strong, well-planned exercise program.

**Petker et al. (2021)**<br><br>**Canada**	RCT	*N* = 124	All	*N* = 59	-	Residential	All	To evaluate an adjunctive yoga program in an inpatient SUD treatment program for adult women.
**Rawson et al. (2015a)**<br><br>**USA**	RCT	*N* = 135	*N* = 40	*N* = 66	a) Methamphetamine dependence per DSM-IV.	Residential	Methamphetamine	The primary aim of this study was to characterize the effects of an 8-week exercise intervention on methamphetamine use outcomes at 1-, 3-, and 6-month post-discharge from residential treatment, compared to a health education control group.
**Rawson et al. (2015b)**<br><br>**USA**	RCT	*N* = 135	*N* = 40	*N* = 66	a) Methamphetamine dependence per DSM-IV.	Residential	Methamphetamine	This study sought to examine the impact of an 8-week structured exercise intervention on reducing depression and anxiety symptoms among a newly abstinent sample of methamphetamine dependent adults compared to a health education condition.

**Roessler (2010)** **Denmark**	Pre-post	*N* = 38	*N* = 15	N/A	a) Adult addicts.	Inpatient and outpatient	Cannabis, opiates, medicine, amphetamines, cocaine and heroin	To describes the experience and outcome of physical exercise used in ambulant treatment of drug addicts and presents the results of that intervention.
**Roessler et al. (2017)** **Denmark**	RCT	*N* = 172	*N* = 52	*N* = 52 (TAU) *N* = 59 (individual physical exercise)	a) Fulfilling ICD-10 criteria for harmful use of or dependence on alcohol.	Outpatient	Alcohol	To examine whether physical activity as an adjunct to outpatient alcohol treatment has an effect on alcohol intake at six months and 12 months after treatment initiation, in particular, the amount and frequency of alcohol intake, including a number of heavy drinking days
**Salem et al. (2022)** **USA**	RCT	*N* = 138	*N* = 40	*N* = 66	a) Methamphetamine dependence diagnosis per DSM-IV	Residential	Methamphetamine	To examine the effectiveness of an 8-week exercise intervention for reducing methamphetamine craving compared to a health education control and the extent to which such differences impact follow-up

								methamphetamine use.
**Sinyor et al. (1982)**<br><br>**Canada**	RCT	*N* = 79	*N* = 24	*N* = 12 (TAU)<br><br>*N* = 9 (lower intensity)	-	Inpatient	Alcohol	To determine whether alcoholics who participate in a graded exercise program show adaptations like those seen in normal and to evaluate whether participation in a fitness program improves subsequent abstinence rates.
**Trivedi et al. (2017)**<br><br>**USA**	RCT	*N* = 302	*N* = 121	*N* = 150	a) Self-reported stimulant use (cocaine, methamphetamine, amphetamine, or other stimulants, excluding caffeine and nicotine) in the 30 days prior to treatment admission, b) met past year DSM-IV criteria for stimulant abuse or dependence.	Residential and outpatient	All	To examine the efficacy of an aerobic exercise intervention in reducing stimulant use by recruiting patients in a residential treatment facility but followed in outpatient treatment settings.
**Unhjem et al. (2016)**	RCT	*N* = 16	*N* = 3	*N* = 7	a) Diagnosed with SUD, classified	Residential	Amphetamine as their primary drug	To assess if a maximal strength training intervention was feasible for

**Norway**					within ICD10: F10-F19			substance use disorder patients and would yield the previously documented beneficial physical and mental effects of such a training regime.
**Vingren et al. (2018)**<br><br>**USA**	RCT	*N* = 16	N/A	*N* = 8	-	Inpatient	All	To determine the effect of resistance training on muscle health markers (mass, strength, and power) and basal circulating biomarkers for men living with HIV undergoing substance abuse treatment.
**Wang et al. (2017)**<br><br>**China**	RCT	*N* = 50	*N* = 6	*N* = 25	(a) DSM-V criteria for drug dependence.	-	Methamphetamine	To determine the effects of aerobic exercise training on craving and inhibition control among people with methamphetamine dependencies.
**Yan-guang et al. (2021)**<br><br>**China**	RCT	*N* = 120	N/A	*N* = 60	a) Diagnosis of methamphetamine dependence according to the DSM-IV Criteria	Rehabilitation Centre	Methamphetamine	To compare the physical fitness of HIIT or MICT intervention amongst individuals with SUD.

**Zhang and Zhu (2020)**<br><br>**China**	RCT	*N* = 76	N/A	*N* = 38	a) Meeting the “guidelines for diagnosis and treatment of amphetamine dependence” issued by the Ministry of Health of the People’s Republic of China in 2009 and the evaluation criteria of addiction syndrome in “Chinese Classification and Diagnostic Criteria of Mental Disorders” (CCMD-3)	Center	Amphetamine	To explore the rehabilitation effect of Taijiquan exercise on patients with amphetamine dependence.
**Zhao et al. (2021)**<br><br>**China**	RCT	*N* = 69	N/A	*N* = 21	a) methamphetamine use disorder residing at the Rehabilitation Center	Center	Methamphetamine	To use the dot-probe task and event-related potential components to investigate the hypothesis that exercise would have a positive effect on attentional bias in individuals with substance use disorder.
**Zhu et al. (2016)**<br><br>**China**	RCT	*N* = 59	N/A	*N* = 29	a) Level 3 illicit drug-dependent users, b) Amphetamine-type	-	Amphetamine	To assess the effect of Tai Chi on the quality of life and fitness in

					stimulant dependents.			individuals with stimulant dependence
**Zhu et al. (2021)**<br><br>**China**	RCT	*N* = 83	N/A	*N* = 41	a) meeting the Diagnostic of DSM-V, screening criteria	Center	Methamphetamine	To investigate the effects of group-based aerobic exercise in promoting the cognitive functions and emotional states of SUD patients.
**Zhu et al. (2018)**<br><br>**China**	RCT	*N* = 80	All	*N* = 38	a) Level 3 illicit drug-dependent users, b) stimulant dependence.	-	Amphetamine	To assess the effect of Tai Chi intervention on female Amphetamine-Type stimulants dependents for sleep quality and fitness change at Shanghai Mandatory Detoxification and Rehabilitation Center.
**Zhuang et al. (2013)**<br><br>**China**	RCT	*N* = 75	All	*N* = 38	a) Having a<br><br>negative urine test for heroin, b) receiving at least 6 months of scheduled therapy at hospital.	-	Heroin	To evaluate whether yoga practice may improve the quality of life and mood status of female heroin abusers under detoxification treatment.

*RCT = Randomized controlled trial; **TAU = Treatment as usual
