# Supplementary material for: Characteristics and impact of physical activity interventions during substance use disorder treatment excluding tobacco: A systematic review
Source: PLoS One. 2023 Apr 26;18(4):e0283861. doi: 10.1371/journal.pone.0283861 (PMC10132651; doi:10.1371/journal.pone.0283861)
Supplement: S4 Table — (PDF) [file pone.0283861.s005.pdf]

S4 Table. Quality assessment tool for randomized trial (K = 35).

Authors	Randomization process	Deviations from intended interventions	Missing outcome data	Measurement of the outcomes	Selection of the reported results	Overall
**Abatti Martin et al. (2017)**	Low	Some concerns	Low	Some concerns	Low	Some concerns
**Brown et al. (2014)**	Some concerns	Some concerns	Low	High	Some concerns	High
**Capodaglio et al. (2009)**	High	High	High	Low	Some concerns	High
**Carmody et al. (2018)**	Some concerns	High	Low	Low	Low	High
**Dolezal et al. (2013)**	Some concerns	High	Low	Some concerns	Low	High
**Ermalinski et al. (1997)**	Some concerns	High	High	High	Low	High
**Flemmen et al. (2014)**	Some concerns	Low	Low	Some concerns	Low	Some concerns
**Gaihre and Rajesh (2017)**	Some concerns	High	Some concerns	Low	Low	High
**Gary and Guthrie (1972)**	Some concerns	High	High	Low	Some concerns	High
**Giesen et al. (2016)**	Some concerns	High	Low	Some concerns	Low	High
**Haglund et al. (2015)**	Some concerns	Low	Low	High	Low	High
**Hallgren et al. (2014)**	Some concerns	Some concerns	Low	High	Low	High
**Li et al. (2013)**	High	High	Low	Low	Low	High
**Liu et al. (2021)**	Some concerns	Some concerns	Low	Low	Low	Some concerns
**Lu et al. (2021)**	Some concerns	Some concerns	Low	Low	Low	Some concerns
**McCartney et al. (2021)**	Some concerns	High	Low	Low	Low	High
**Nygard et al. (2018)**	Some concerns	High	Low	Low	Low	High

**Palmer et al. (1988)**	High	High	High	High	Low	High
**Petker et al. (2021)**	High	Low	Some concerns	Some concerns	Low	High
**Rawson et al. (2015a)**	Some concerns	Low	Low	Low	Low	Some concerns
**Rawson et al. (2015b)**	Some concerns	Some concerns	Low	High	Low	High
**Roessler et al. (2017)**	Low	Low	Low	Some concerns	Low	Some concerns
**Salem et al. (2022)**	Low	Low	Low	Low	Low	Low
**Sinyor et al. (1982)***	High	High	High	Low	Some concerns	High
**Trivedi et al. (2017)**	Low	Low	Low	Low	Low	Low
**Unhjem et al. (2016)**	Some concerns	High	Low	Some concerns	Low	High
**Vingren et al. (2018)**	Some concerns	High	Low	Low	Low	High
**Wang et al. (2017)**	Some concerns	High	High	Low	Low	High
**Yan-guang et al. (2021)**	Some concerns	High	Some concerns	Low	Low	High
**Zhang et al. (2020)**	Some concerns	Some concerns	Low	Low	Low	Some concerns
**Zhao et al. (2021)**	Some concerns	High	High	Low	Low	High
**Zhu et al. (2016)**	High	Some concerns	Low	High	Low	High
**Zhu et al. (2018)**	High	High	Low	Low	Low	High
**Zhu et al. (2021)**	Some concerns	Low	Low	Low	Low	Some concerns
**Zhuang et al. (2013)**	Low	Some concerns	Low	High	Low	High

* = Cluster.
