# Supplementary material for: Characteristics and impact of physical activity interventions during substance use disorder treatment excluding tobacco: A systematic review
Source: PLoS One. 2023 Apr 26;18(4):e0283861. doi: 10.1371/journal.pone.0283861 (PMC10132651; doi:10.1371/journal.pone.0283861)
Supplement: S6 Table — (PDF) [file pone.0283861.s007.pdf]

S6 Table. Quality assessment tool for before-after (pre-post) studies with no control group (K =6).

Reference	1	2	3	4	5	6	7	8	9	10	11	12	Overall
**Brown et al. (2009)**	Y	Y	N	N	N	Y	Y	NA	N	Y	Y	NR	Fair
**Brown et al. (2010)**	Y	Y	N	Y	N	Y	Y	NR	N	Y	Y	NR	Fair
**Frankel and Murphy (1974)**	N	N	Y	NR	NR	Y	Y	NR	NR	Y	N	NR	Poor
**Muller and Clausen (2015)**	Y	Y	Y	NR	N	Y	Y	N	Y	Y	N	NR	Fair
**Ness et al. (2001)**	Y	N	Y	NR	N	Y	Y	NR	Y	Y	N	NR	Fair
**Roessler (2010)**	Y	N	N	NR	N	N	Y	NR	N	Y	N	NR	Poor

Y = Yes; N = No; NA= Not applicable; NR = Not reported; 1 = Was the study question or objective clearly stated?; 2 = Were eligibility/selection criteria for the study population prespecified and clearly described?; 3 = Were the participants in the study representative of those who would be eligible for the test/service/intervention in the general or clinical population of interest?; 4 = Were all eligible participants that met the prespecified entry criteria enrolled?; 5 = Was the sample size sufficiently large to provide confidence in the findings?; 6 = Was the test/service/intervention clearly described and delivered consistently across the study population?; 7 = Were the outcome measures prespecified, clearly defined, valid, reliable, and assessed consistently across all study participants?; 8 = Were the people assessing the outcomes blinded to the participants' exposures/interventions?; 9 = Was the loss to follow up after baseline 20% or less? Were those lost to follow-up accounted for in the analysis?; 10 = Did the statistical methods examine changes in outcome measures from before to after the intervention? Were statistical tests done that provided p values for the pre-to-post changes?; 11 = Were outcome measures of interest taken multiple times before the intervention and multiple times after the intervention (i.e., did they use an interrupted time-series design)?; 12 = If the intervention was conducted at a group level (e.g., a whole hospital, a community, etc.) did the statistical analysis take into account the use of individual-level data to determine effects at the group level
