# Supplementary material for: Characteristics and impact of physical activity interventions during substance use disorder treatment excluding tobacco: A systematic review
Source: PLoS One. 2023 Apr 26;18(4):e0283861. doi: 10.1371/journal.pone.0283861 (PMC10132651; doi:10.1371/journal.pone.0283861)
Supplement: S7 Table — (PDF) [file pone.0283861.s008.pdf]

S7 Table. Characteristics of the physical activity interventions.

Authors	Frequency by week	Duration (weeks)	Intensity	Activity	Time duration (minutes)	Amount (Frequency X time)
**Abatti Martins et al. (2017)**	3	12	Light	Resistance exercise	-	-
**Brown et al. (2009)**	1	12	Moderate	Jogging, cycling, elliptique	20 - 40	20 - 40
**Brown et al. (2010)**	1	12	Moderate	Jogging, cycling, elliptique	30	30
**Brown et al. (2014)**	1	12	Moderate	Jogging, cycling, elliptique	20 - 40	20 - 40
**Burling et al. (1992)**	3	-	Moderate*	Softball	-	-
**Capodaglio et al. (2003)**	-	2	Vigorous	Walking, cycling	50	-
**Carmody et al. (2018)**	3	12	Moderate	Jogging	-	-
**Dolezal et al. (2013)**	3	8	Vigorous	Resistance exercise	60	180
**Ermalinski et al. (1997)**	5	6	Light and moderate*	Yoga, jogging	20	100
**Fitzgerald et al. (2020)**	2	-	Light*	Yoga	-	-
**Flemmen et al. (2014)**	3	8	Vigorous	Jogging	28	84
**Frankel and Murphy (1974)**	5	12	Moderate*	Resistance exercise, aerobic	60	300
**Gaihre and Rajesh (2017)**	6	12	Light and moderate	Yoga, jogging	90	540
**Gary and Guthrie (1972)**	5	4	Moderate*	Jogging	-	-
**Giesen et al. (2016)**	2	52	Moderate and vigorous	Cycling, resistance exercise, event	60	120
**Haglund et al. (2014)**	3	8	Moderate*	aerobic, resistance exercise	60	180

**Hallgren et al. (2014)**	1	10	Light	Yoga	90	90
**Li et al. (2013)**	4	24	Moderate	Tai chi	80	320
**Liu et al. (2021)**	5	12	Moderate	Aerobic exercise	40	200
**Lu et al. (2020)**	3	12	Moderate	Cycling	40	120
			Moderate*	Anaerobic resistance exercise	-	-
**McCartney et al. (2020)**	6	1	Moderate	Cycling	35	210
**Muller and Clausen (2015)**	3	10	Light	Jogging, resistance exercise, ball game	30	90
**Ness et al. (2001)**	3	6	Moderate*	Aerobic, resistance exercise	-	-
**Nygard et al. (2018)**	3	12	Moderate*	Resistance exercise	-	-
**Palmer et al. (1988)**	3	4	Moderate	Jogging, walking	25	75
**Petker et al. (2021)**	3	5	Light*	Yoga	60	180
**Rawson et al. (2015a)**	3	8	Moderate	Jogging, resistance exercise	55	165
**Rawson et al. (2015b)**	3	8	Moderate	Jogging, resistance exercise	55	165
**Roessler (2010)**	3	8	Moderate*	Sport, cycling, resistance exercise	120	360
**Roessler et al. (2017)**	2	24	Moderate*	Jogging	-	-
**Salem et al. (2022)**	3	8	Moderate*	Progressive aerobic, resistance exercise	60	180
**Sinyor et al. (1982)**	5	6	Vigorous	Jogging	60	300
**Trivedi et al. (2017)**	3	12	Moderate	Walking	-	-
**Unhjem et al. (2016)**	3	8	Moderate*	Resistance exercise	-	-

**Vingren et al. (2018)**	3	6	Moderate*	Resistance exercise	-	-
**Wang et al. (2017)**	3	12	Moderate	Cycling, jogging, jump rope	40	120
**Yan-guang et al. (2021)**	3	52	High	HIIT (resistance training, running)	60	180
			Moderate	Tai chi, mind-body, recreational activity		
**Zhang and Zhu (2020)**	5	24	Light*	Taijiquan	50	250
**Zhao et al. (2021)**	3	12	Moderate	Cycling	40	120
			High			
**Zhu et al. (2016)**	5	12	Moderate	Tai chi	50	250
**Zhu et al. (2021)**	5	12	Moderate	Aerobic and resistance exercise	36	180
**Zhu et al. (2018)**	4	24	Moderate	Tai chi	60	240
**Zhuang et al. (2013)**	5	24	Light*	Yoga	50	250

*Determined by the authors.
