# Supplementary material for: Characteristics and impact of physical activity interventions during substance use disorder treatment excluding tobacco: A systematic review
Source: PLoS One. 2023 Apr 26;18(4):e0283861. doi: 10.1371/journal.pone.0283861 (PMC10132651; doi:10.1371/journal.pone.0283861)
Supplement: S8 Table — (PDF) [file pone.0283861.s009.pdf]

S8 Table. Outcomes measure, main results and bias.

Authors and years	Outcomes measures (assessment method when available)	Main results	Bias
**Abatti Martins et al. (2017)**	Anthropometry<br><br>(Body weight, percentage of total body fat, fat body mass, body mass index, lean body mass; waist/hip ratio)	Lower percentage of fat body mass for the exercise group post-intervention.	Cochrane: Some concerns
	Physical fitness and health<br><br>(cardiorespiratory capacity, muscle force/resistance test [abdominal and arm flexion/extension], abdominal test, length of the jump, lumbar traction, handgrip test, trunk flexibility)	Higher flexibility, abdominal strength, handgrip strength and cardiorespiratory capacity for the exercise group post-intervention.<br><br>There was a reduction in upper limb strength, abdominal strength and VO_2 max post-intervention for the sedentary group.	
**Brown et al. (2009)**	Alcohol and drug use<br><br>(TLFB)	Significant increases in percent days abstinent were observed at the end of the 12-week exercise intervention and at the 3-month post-intervention follow-up. Decreased drinks per drinking day at end of treatment and a significant reduction in drinks per drinking day at 3-months postintervention compared to baseline levels of drinking.	
	Cardiorespiratory fitness<br><br>(Submaximal graded exercise protocol on a motorized treadmill)	Participants significantly improved on the duration of the submaximal treadmill test at end of treatment.	
	Body composition<br><br>(Weight, body fat percentage, BMI)	No significant differences.	
**Brown et al. (2010)**	Alcohol and drug use<br><br>(TLFB)	In terms of percent days abstinence for drug use, there was a significant increase at the end of treatment and a trend toward increased 3-month post-intervention.	NIH: Some concerns

		At the end of the exercise intervention, 66.7% of the sample did not relapse and had been continuously abstinent.<br><br>Non-attenders of the exercise intervention were significantly more likely to relapse than attenders.	
	Cardiorespiratory fitness<br><br>(Submaximal graded exercise protocol on a motorized treadmill)	Improving the duration of the submaximal treadmill test at the end of treatment and significant improvement in cardiorespiratory fitness at the 3-month post-intervention follow-up.<br><br>In addition, compared to the baseline, participants were able to reach 85 % of their maximal heart rate at higher MET levels at the 3-month follow-up.	
	Body composition<br><br>(Weight, body fat percentage, BMI)	No significant differences.	
**Brown et al. (2014)**	Alcohol use<br><br>(TLFB)	Participants who were adherent to exercise intervention had significantly fewer drinking and heavy drinking days than those in brief advice to exercise comparison intervention during treatment.	Cochrane:
	Depressive symptoms<br><br>(CES-D)	No significant differences.	
	Anxiety symptoms<br><br>(STAI)	No significant differences.	
	Self-efficacy for alcohol abstinence<br><br>(Situational Confidence Questionnaire)	No significant differences.	
	Level of exercise<br><br>(TLFB)	Adherent participants in exercise group reported greater minutes of exercise than brief advice to exercise comparison intervention during treatment. No such difference was observed after the 12-week follow-up.	

	Physical Fitness<br><br>(Submaximal graded exercise protocol on a motorized treadmill)	VO2 max increased significantly more among those adherent to exercise group compared to those in brief advice to exercise comparison intervention.	
**Burling et al. (1992)**	Length of stay	The softball group remained in treatment significantly longer and more likely to complete the inpatient than both control groups.	NIH: High
	Living situation<br><br>(Interview)	The softball group compared to the control group, had higher drug-alcohol abstinence rates and were more likely to have been employed and stably housed.	
	Employment status<br><br>(Interview)		
	Drug/alcohol use<br><br>(Interview)		
**Capodaglio et al. (2003)**	Physiological variables<br><br>(Heart rate, blood pressure)	Baseline heart rate was significantly lower in the healthy group as regards the patients, in both sessions.<br><br>No differences for the blood pressure value.	Cochrane: High
	Work capacity (three submaximal trials : lifting, walking, and arm-cranking)	Walking: Velocity slightly increased in the exercise group reached comparable to the healthy group. Chosen velocity is similar in the exercise group and the healthy group. The Exercise group (not significant) and the TAU group (significant) showed a reduced exercise duration compared to the healthy group.<br><br>Lifting: The exercise group showed a significant improvement in total work. The exercise group chose in both sessions heavier weights compared to the healthy group<br><br>Arm cranking: The selected workloads were similar between all groups in both sessions, but exercise duration was significantly shorter in the exercise group. Perceived exertion was similar between groups.	

		Work capacity: The exercise group consistently showed an increase in functional work capacity after training: subjects were in fact able to perform a greater quantity of aerobic work with no changes in perceptual level.	
**Carmody et al.**<br><br>**(2018)**	Drug use<br><br>(TLFB, UDS)	Significant group effect for the probability of use in the groups.<br><br>Significant group effect for the days of use among those who used.	Cochrane: High
	Attendance<br>(Treatment tracking)	The exercise group experienced a significantly lower relapse rate than the health education group and once a participant relapse, the days of use were significantly lower in the exercise group compared to the health education group.	
**Dolezal et al. (2013)**	Anthropometry<br>(Body weight, body composition and density, relative body fat)	The exercise group significantly reduced percent relative body fat and fat weight with these differences also significant between groups.	Cochrane: High
	Aerobic performance<br><br>(cardiopulmonary exercise test, aerobic capacity, 12-lead electrocardiogram)	The exercise group increased maximum oxygen uptake while this measure did not change in the equal attention education group.	
	Musculoskeletal fitness<br><br>(muscle strength and endurance)	The exercise group increased lower body strength, upper body strength and it was significant between the 2 groups.	
**Ermalinski et al. (1997)**	Physical fitness<br><br>(cardiovascular capacity, muscular flexibility)	The exercise group has a greater flexibility to stretch their back.<br><br>The exercise group improved significantly more than those in the TAU groups in systolic blood pressure and in double product.	Cochrane: High
	Responsibility for health (multidimensional health locus of control scales)	The exercise group saw themselves as having more of an internal locus of control and being less controlled by powerful other than the TAU group.<br><br>Within-group comparisons showed that the TAU group did not change their evaluation of the locus of control as did the exercise group.	

	Body satisfaction<br><br>(long and short form of the body cathexis)	No significant differences in total body satisfaction score.<br><br>The exercise group expressed significantly greater satisfaction than the TAU group with their breathing, energy level, and body build.	
	Self-esteem<br><br>(self-cathexis scale)	No significant differences	
	Depression<br><br>(Depression adjective check list)	No significant differences	
	Ratings of therapy groups<br><br>(satisfaction with sleep, satisfaction with therapy group, attractiveness of therapy, belonging to a therapy group, craving for alcohol)	There was no between groups differences in the five ratings.<br><br>The exercise group had a significantly greater decrease in craving for alcohol than the TAU group.	
**Fitzgerald et al. (2020)**	Mood states<br><br>(POMS-ASF)	Significant differences between the pre-and post-yoga session scores for all but the Vigor mood state for both genders.	NIH: Some concerns
**Flemmen et al.**<br><br>**(2014)**	Maximal Oxygen Consumption and Work Economy	Both groups improved their VO_2max.<br><br>The exercise group increased velocity and inclination to VO_2max from pre-to post-test.<br><br>The exercise group increased ventilation at VO_2max but no differences within or between groups in respiratory exchanged ratios and in lactate concentration at VO_2max from pre-to post-test.<br><br>The TAU group showed no within-group improvement in neither VO_2 max nor maximal workload.<br><br>Work economy showed no significant differences between or within the two groups following the training period.	Cochrane: Some concerns

		Heart rate in the work economy workload significantly decreased in the exercise group.	
	Insomnia<br><br>(ISI)	No significant difference	
	Anxiety and Depression<br><br>(HAD)	The exercise group decreased in depression levels at the post-test whereas the TAU group decreased in anxiety from pre-to post-test. (No between groups difference)	
**Frankel and Murphy (1974)**	Physical fitness<br><br>(Weight, resting pulse, systolic blood pressure, diastolic pressure, a submaximal step test, modified version of Illinois standard test)	All the changes from entry to exit were statistically significant except the decrease in systolic blood pressure.	NIH: High
	Personality<br><br>(The standard 13 scales of the Minnesota multiphasic personality inventory)	At the entry, the profile was 2-4-7 and, at the exit, the profile was a normal K.<br><br>Greatest diminution was in neurotically anxious depressive affect.<br><br>15/19 variables showed significant changes (personality and physical fitness).	
**Gaihre and Rajesh (2017)**	Working memory and executive function<br><br>(Stroop test, digits forward, digits backward, and the cancellation task)	No significant differences.	Cochrane: High
**Gary and Guthrie (1972)**	Personality<br><br>(Gough adjective checklist)	No significant differences.	Cochrane: High
	The Jourard body cathexis	Positive physical fitness and masculinity scale of the Gough adjective checklist, change in total physical fitness and change in personality	

		variables (body-cathexis and self-cathexis) and negative (self-criticality) correlations.	
	Self-cathexis scale	Only Self-cathexis scale improved significantly.	
	Schneider physical test	The exercise group improved the score of Scheider (average) and control group stays at (poor).	
	Drinking behavior	No significant differences.	
	Sleep disturbances	The exercise group showed a significant lower level of sleep disturbances.	
**Giesen et al. (2016)**	Physical activity level<br><br>(sensory armbands)	Significant increases in kcal per week and steps per week for the exercise group and decreased values in the control group.	Cochrane: High
	Health-related quality of life<br><br>(SF-36)	Significant improvements for the exercise group regarding 4 out of the 8 SF-36 (physical functioning, general health perceptions, vitality, and mental health)	
	Relapse<br><br>(Alcohol and drug use, Breathalyzer and/or urine test)	In the exercise group no one relapse, control group 4 relapse and 3 of the drop-out candidate.	
**Haglund et al. (2014)**	Symptoms of depression (BDI)	The exercise group significantly greater reduction in depression symptom (BDI) scores than the education groups.<br><br>Participants who attended the greatest amount of exercise sessions derived the greatest benefit.	Cochrane: High
**Hallgren et al. (2014)**	Alcohol use (TLFB)	No significant differences.	Cochrane: High
	Alcohol dependence (presence of biomarkers: GT, CDT)	No significant differences.	
	Depression and anxiety (HAD)	No significant differences.	

	Health-related functioning and quality of life (Sheehan Disability scale)	No significant differences.	
	Stress<br><br>(Perceived Stress Scale, sample of saliva for cortisol levels)	No significant difference.	
**Li et al. (2013)**	Physiological parameter<br><br>(Blood sample)	No significant difference<br><br>However, the difference of B lymphocytes tended to be significant between the exercise group and the control group over the course of Tai Chi training.	Cochrane: High
	Rating Scale of Heroin Withdrawal Symptoms	The exercise group and the drop-out group were statistically different only in 60 days.	
	Depression<br><br>(Hamilton Rating Scale for Depression)	The exercise group and the drop-out group were statistically different only at 60 days.	
**Liu et al. (2021)**	Attention and Working Memory<br><br>(Digit Span Test, Trail Making Test)	Significantly higher in the study group than the control group.	Cochrane:
	Verbal Memory<br><br>(Logical Memory, Memory for Persons Data)	The study group had significantly better post-test in Memory for Persons Data scores than the control group. No significant differences in scores between the two groups were found for logical memory.	
	Executive Function<br><br>(Color-Word Stroop Test)	The effect of aerobic exercise on executive function was dramatically greater in the study group than in the control group.	
**Lu et al. (2020)**	Brain activity<br><br>(Electroencephalography)	The mean frequency of the frontal, temporal, and occipital regions in drug-cue condition were decreased after 3 month exercise treating especially for patients with resistance exercise program while the control group showed increase.	
	Craving	No significant difference.	

	(Visual analog scale)		
**McCartney et al. (2020)**	Sleep behaviour<br>(Actiwatch)	Overall, results indicate that compared to stretching, cycling had a positive effect on sleep duration, sleep efficiency and average wake bout during the Treatment phase. These improvements were not reflected in participants' subjective score.	
	Sleep quality<br>(Modified sleep condition indicator questionnaire)		
	Insomnia<br>(ISI)		
**Muller and Clausen (2015)**	Quality of life<br>(WHOQOL-BREF)	Time X Exercise interaction:<br>- Statistically significant in the physical health domain with an increase in the completers (above minimum clinically important difference threshold) and a decrease in the non-completers.<br>- In the psychological health domain with an increase in the completers (main effect of time too).	NIH: Some concerns
	Mental distress<br>(HSCL-25)	No significant difference.	
	Substance-related variables<br>(European Addiction Severity Index)	No significant difference.	
	Somatic health burden<br>(List of 25 conditions)	Unchanged in both groups.	
	Feasibility<br>(the attendance data)	Participants exercised for an average of 13.2 sessions out of 30.	
	Strength	No significant difference.	

**Ness et al. (2001)**	(upper and lower body: 1 RM, handgrip)		NIH: Some concerns
	Aerobic fitness<br><br>(Heart rate after and before exercise, continuous exercise on an ergometer or treadmill)	No significant difference.	
	Balance<br><br>(Berg Balance Measure)	Significant difference for balance.	
	Functional performance<br><br>(The Physical Performance Test)	Significant improvement of functional performance.	
**Nygard et al. (2018)**	Maximal muscle strength and rate of force development<br><br>(1RM using a hack-squat apparatus angled 45° to the vertical)	The exercise group improved half-squat 1RM and rate of force and peak force more than the control group.	Cochrane: High
	Skeletal health<br><br>(bone mineral content and density, body composition, blood sample)	The exercise group increased lean mass and whole body.<br><br>The exercise group increased bone mineral content in whole body, lumbar spine, femoral neck (no change in the control group but improvement in the lumbar spine was apparent as a between-groups difference).<br><br>The trend toward improvement in total hip in the exercise group was apparent as a between-group difference.<br><br>The exercise group improved t-score at the lumbar spine, trochanter, intertrochanteric hip (between group differences).<br><br>The trabecular bone score did not change in either of the two groups from pre-to post-test.<br><br>No changes were observed in serum levels of P1NP, CTX, or 25-hydroxy vitamin D3 in either of the two groups.	

	Anxiety, depression<br><br>(HAD)	The exercise group exhibited a tendency to improve levels of anxiety and depression more than the control group.	
	Insomnia<br><br>(ISI)	-	
**Palmer et al. (1988)**	Depression<br><br>(Zung Self-Rating Depression Scale)	Significant difference.	Cochrane: High
	Anxiety<br><br>(Spielberger State-Trait Anxiety Inventory)	Significant difference in state anxiety and trait anxiety.	
	Self-concept<br><br>(Tennessee Self-Concept Scale)	No significant difference.	
	Estimated maximum oxygen uptake<br><br>(Astrand-Rhyming bicycle-ergometer test)	No significant difference.	
**Petker et al. (2021)**	Anxiety and PTSD symptoms<br><br>(PHQ and PTSD Checklist for DSM-5)	Difference pre-post but not between group	
	Physical symptoms<br><br>(PHQ)	Main effect of time for yoga group	
	Drug cravings<br><br>(Penn Craving Scale)	Significant main effect of time for both group	
	Mindfulness<br><br>(UPPS-P Impulsive Behavior Scale)	Significant main effect for yoga group	

**Rawson et al. (2015a)**	Methamphetamine use<br><br>(UDS, Substance Use Inventory)	Lower severity in the exercise group were significantly less likely to use MA at 1, 3, and 6 months post-discharge compared to lower severity in education group.	Cochrane : Some concerns
**Rawson et al. (2015b)**	Depression<br><br>(BDI)	The exercise control group showed lower depression and anxiety total scores than the education group at the 8-week time point from the baseline.<br><br>A significant dose interaction effects between session attendance and exercise were found as well on reducing depression and anxiety symptoms over time compared to the control group.	Cochrane: High
	Anxiety<br><br>(Beck Anxiety Inventory)		
**Roessler (2010)**	Physical fitness<br><br>(maximal oxygen intake)	The results of the participants who completed the program showed a significant increased in oxygen uptake	NIH: High
	Addiction behavior in the short and longer term<br><br>(European Addiction Severity Index)	The long-term effect showed that five of the 20 abusers interviewed reported that they still had not taken drugs, 10 had downgraded their intake, four experienced no change at all.	
	Body image and self-confidence<br><br>(interview)	The addicts obtained a better body image, became more sensitive to physical pain and disorders and reduced their drug intake during the training period. They also said that fitness reduced suffering from withdrawal and their new quality of life.	
**Roessler et al. (2017)**	Alcohol intake<br><br>(TLFB)	At 6-month follow-up: all groups showed a marked reduction in alcohol intake.<br><br>No difference between groups concerning drinking outcomes measured as consumed by units of alcohol per month.<br><br>The number of days of abstinence had increased while the number of drinks per drinking day had decreased across the total sample.<br><br>No significant difference for excessive drinking.	Cochrane: High

	Physical activity<br><br>(IPAQ)	Moderate physical activity levels compared to low levels: protective effect on drinking behavior.<br><br>Moderate levels of physical activity had lower odds for excessive drinking and higher abstinence rate than participants with low-level physical activity.	
**Salem et al. (2022)**	Craving and use for methamphetamine<br><br>(Visual Analog Scale, USD and Substance Use Inventory)	Participants in the control group had a significantly higher total mean weekly craving score during the 8-week trial, compared to the exercise condition.	
**Sinyor et al. (1982)**	Fitness<br><br>(percentage of body fat, heart rate, maximal oxygen uptake)	The exercise group significantly reduced the percentage of body fat, significantly increased maximal oxygen uptake and showed a trend to decrease basal heart rate.<br><br>The exercise group with lower intensity failed to show the types of changes across test sessions.	Cochrane: High
	Abstinence rates	The exercise group with higher intensity and the exercise group with lower intensity improved abstinence rates better than the control group (18-month follow-up).	
**Trivedi et al. (2017)**	Use of drugs<br><br>(TLFB, UDS)	No significant difference.	Cochrane: Some concerns
**Unhjem et al. (2016)**	Strength<br><br>(1 RM hack squat and plantar flexion, peak force, rate of force development)	After 8-week the exercise group increased 1 RM hack squat, plantar flexion, rate of force development and peak force. Control group showed no changes.	Cochrane: High
	Muscle activation<br><br>(The V-wave method)	Enhanced efferent neural drive in the exercise group.<br><br>The exercise group increased m. soleus V_{max}. when the control group showed no changes.	

	Psychological<br><br>(HAD, ISI)	Both groups displayed significant within group reductions in anxiety levels, a tendency to decrease the level of depression, while the level of insomnia significantly decreased only in the exercise group.	
**Vingren et al. (2018)**	Strength<br><br>(muscle mass, upper-body and lower-body muscle strength, maximal lower-body power, and resting and fasted concentrations of a panel of circulating cytokines, vascular cellular adhesion molecule–1 [VCAM-1], and cortisol).	The exercise group increased significantly their measures of strength and lower-body power from pre-to post-intervention and compared to the control group.	Cochrane: High
	Anthropometric measures<br><br>(body mass, height, skinfolds, body segment circumferences, muscle mass, upper-arm circumference, forearm circumference, skinfold thickness)	Significant increased in muscle mass and arm circumference for the exercise group but unchanged in the control group.	
**Wang et al. (2017)**	Craving<br><br>(visual analog scale)	Lower scores of cravings in the exercise group compared to the attentional control group from 6 to 12 weeks.	Cochrane: High
	Inhibitory control<br><br>(Standard Go/Nogo and MA-related Go/Nogo tasks, electroencephalographic)	Standard go/nogo task: the exercise group was higher than the attentional control group in post-test and both groups post-test was higher.<br><br>Ma-dependant go/nogo task: the exercise group during post-test had greater accuracy than pre-test for both go-ma and go-neutral. During post-test, greater accuracy for the exercise group compared with the attentional control group between nogo-ma and nogo-neutral (no observed in go-ma and go-neutral). The go- showed greater accuracy than nogo.<br><br>N2 of neuroelectric (standard go/nogo task): larger n2 amplitudes in the exercise group during post-test compared to pre-test in both go and nogo conditions (largest n2 amplitude was seen in the exercise group during	

		post-test). Nogo condition presented a larger amplitude compared to go condition at both time points.<br><br><u>N2 of neuroelectric (ma-dependant go/nogo task):</u> larger n2 amplitudes in nogo-ma condition during post-test in the exercise group compared to the attentional control group (no observed in nogo-neutral condition). Larger n2 amplitudes were observed in the exercise group during post-test compared to pre-test. Larger n2 amplitude was observed in the nogo-ma condition compared to those in go-ma condition in the exercise group during both time points. Largest n2 amplitudes were observed in fz for all participants compared with those of cz and pz electrodes during both time points.	
**Yan-guang et al. (2021)**	Physical fitness<br><br>(Test: Push-up, sit-and-reach, one-leg standing with eyes closed, choice reaction time and hand grip)	No significant differences were found between groups and interactions of group × time in terms of hand-grip power, push-up, sit-and-reach, one-leg standing with eyes closed and choice reaction time test	
	Physiological<br><br>(HR and BP)	There was no significant difference between groups and interactions of group × time	
	Craving level	The craving level significantly decreased in both groups, but no significant differences with interactions of group × time were found.	
**Zhang and Zhu (2020)**	Physical fitness<br><br>(National Physical Fitness Test tool)	Body composition: the interaction between time and group was not significant<br>Cardiovascular system (Heart rate and blood pressure): the interaction between time and group was not significant<br>Physique: Only significant improvement in balance.	
	Depression and anxiety symptoms<br><br>(Baker self-rating depression scale, state-trait anxiety scale)	No significant difference for depression symptom between group.<br><br>Significant difference at 6 months and the trait anxiety decreased more in Taijiquan group.	
	Quality of life	No significant difference between groups.	

	(SF-36)		
	Craving<br><br>(Amphetamine craving scale)	Interaction between time and group was significant, difference was significant at 3 months and highly significant at 6 months (psychological craving of Taijiquan group was much lower than that of the control group).	
**Zhao et al. (2021)**	Attentional bias index<br><br>(electroencephalography)	Significant interaction between the main effects of group and time. The attentional bias indexes in the high-intensity and moderate-intensity exercise group post exercise were significantly lower than those before exercising.	
	Event-related potential component<br><br>(electroencephalography)	Amplitudes decreased, and the P2 amplitudes changed so that they no longer differed between the congruent and incongruent conditions after 12 weeks of exercise	
**Zhu et al. (2016)**	Quality of life<br><br>(QOL-DAv2.0)	The exercise group improved significantly after 3 months of intervention compared with the slight reduction in the TAU group.<br><br>Although the psychology score of the TAU group showed no significant change.	Cochrane: High
	Fitness<br><br>(Blood pressure, body mass, body composition, upper limb strength, balance, range of motion)	The exercise group showed an improvement in balance control.<br><br>Bilateral handgrip power in the exercise group was significantly higher than in the TAU group.<br><br>No significant change in upper-limb strength<br><br>Blood pressure decreased in both groups.<br><br>Body mass of participants in both groups increased.	
**Zhu et al. (2021)**	Cognitive function<br><br>(Experiment: stop-signal paradigm)	No significant difference	
	Anxiety symptoms<br><br>(Hamilton Anxiety Scale)	Main effect for time and an interaction of group by time.	

	Depression symptoms<br><br>(Beck Depression Inventory-II)	At 3 months, the BDI score of the exercise group was significantly lower than that of the control group	
	Craving<br><br>(Visual Analog Scale)	At 2 and 3 months, the visual analog scores of the exercise group were all significantly smaller than those of the control group	
	Physical fitness<br><br>(BMI, vital capacity, a step test, and grip strength, flexibility, balance, and selection response time)	At 3 months, the physical fitness score of the exercise group was significantly higher than that of the control group.<br><br>The BMI of the exercise group at 3 months was significantly lower than that of the control group. Moreover, the vital capacity and flexibility scores of the exercise group at 1 month, 2 months, and 3 months were all better than those of the control group at those time points. Also, at 2 months and 3 months, the balance scores of the participants in the exercise group were better than the balance scores of those in the control group. No significant differences were found between the two groups, however, for the indicators of stepping, grip strength, and reaction time	
**Zhu et al. (2018)**	Self-related Sleep Quality<br><br>(PSQI)	Between groups showed difference for sleep duration, habitual sleep efficiency (decrease compared to the TAU group), need for sleep medication and PSQI score (decrease compared to the TAU group).<br><br>Time X Group interaction: longer sleep duration for the exercise group. Need for medication and daytime dysfunction different for both groups (decreased).	Cochrane: High
	Depression<br><br>(SDS)	SDS decreased in the exercise group compared to the TAU group.	
	Physical effect<br><br>(Blood pressure, body composition and mass index, handgrip power test, flexibility, balance test, progressive aerobic cardiovascular endurance run)	Time X Group interaction: decreased pulse rate in the exercise group compared to the TAU group.<br><br>Between-groups differences: body fat and running laps of PACER.<br><br>The fitness test results showed an improvement in the balance control but no significant differences between groups.	

		No significant difference between groups in handgrip power test and flexibility.	
**Zhuang et al. (2013)**	Quality of life<br><br>(SF-36)	Rising trends in SF-36 subscales in both groups over time.<br><br>In addition, mean scores of the PCS and MCS increased slightly in the control group and rose significantly in the exercise group over 6 months<br><br>Group effects: role-physical, bodily pain, general health perceptions, vitality, role-emotional, mental health.<br><br>All scores of eight variables revealed a significant difference of time and interaction between time and group.	Cochrane: High
	Mood status<br><br>(POMS)	Group effects over time: tension and anxiety, depression, fatigue and confusion.<br><br>Mean scores of the POMS improved over time in the exercise group.	
